# Supplementary material for: Single-shot two-dimensional nano-size mapping of fluorescent molecules by ultrafast polarization anisotropy imaging
Source: Nat Commun. 2025 May 30;16:5019. doi: 10.1038/s41467-025-60072-1 (PMC12125188; doi:10.1038/s41467-025-60072-1)
Supplement: Supplementary file 1 — Supplementary Information [file 41467_2025_60072_MOESM1_ESM.pdf]

Supplementary Information for

Single-shot two-dimensional nano-size mapping of fluorescent molecules by ultrafast polarization anisotropy imaging

Peng Wang[1,‡], Yogeshwar Nath Mishra[1,2,3,‡], Florian J. Bauer[4,‡], Murthy S. Gudipati[2] and Lihong V. Wang[1,*]

[1]Caltech Optical Imaging Laboratory, Andrew and Peggy Cheng Department of Medical Engineering, Department of Electrical Engineering, California Institute of Technology, 1200 East California Boulevard, Mail Code 138-78, Pasadena, CA 91125, USA

[2]Science Division, Jet Propulsion Laboratory, California Institute of Technology, 4800 Oak Grove Drive, Pasadena, CA 91109, USA

[3]Visual Computing Center, KAUST, Thuwal 23955-6900, Saudi Arabia

[4]Lehrstuhl für Technische Thermodynamik (LTT) and Erlangen Graduate School in Advanced Optical Technologies (SAOT), Universität Erlangen-Nürnberg, Erlangen 91058, Germany

*Corresponding author: LVW@caltech.edu

‡These authors contributed equally to this work

1. **Setup and equipment**

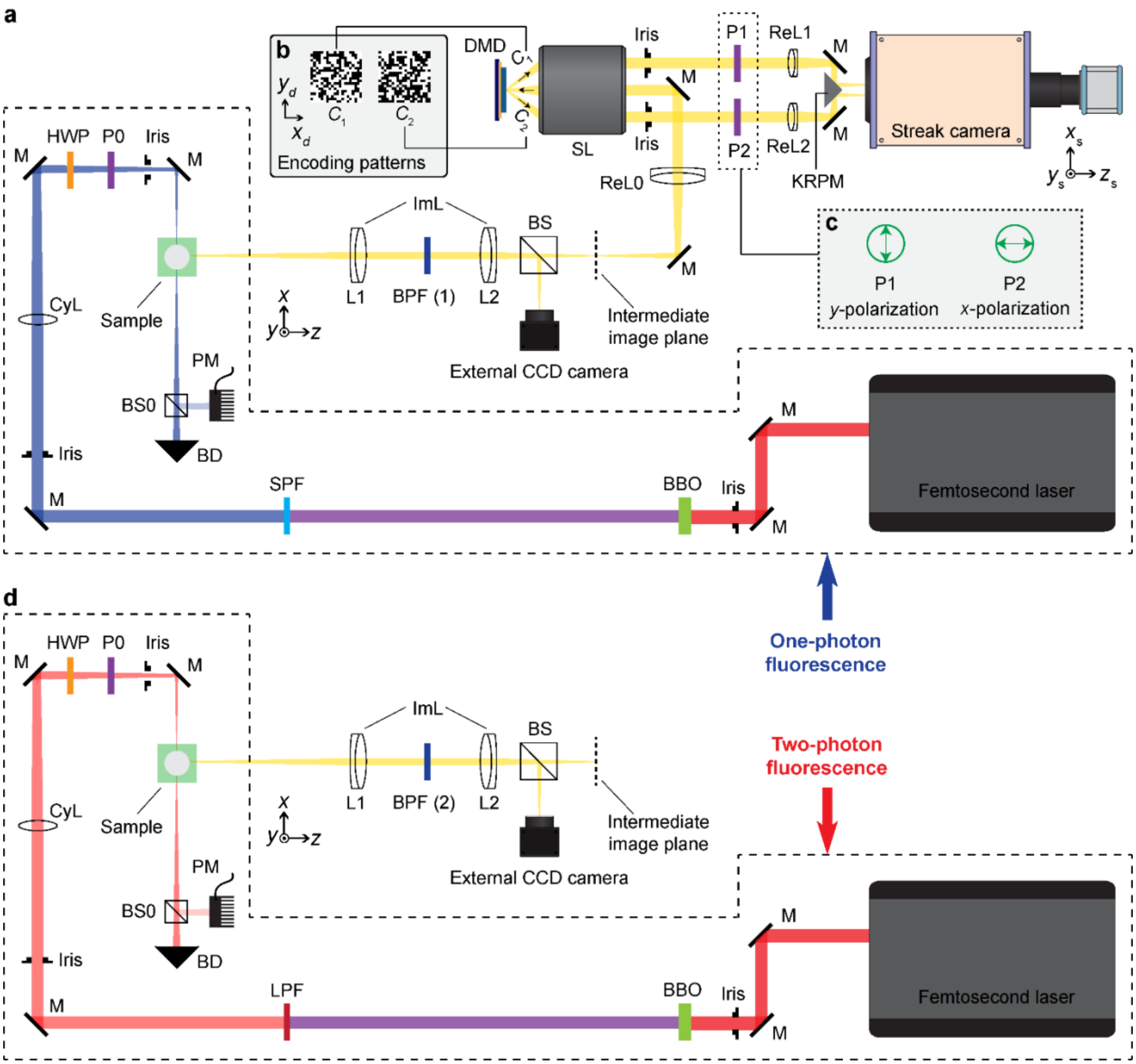

Fig. S1. A detailed schematic of the CUP2AI system for one-photon fluorescence excitation (top) and two-photon fluorescence excitation (bottom). The full names of the abbreviations can be found in Table S1. (**a**), The entire system when imaging one-photon fluorescence (1PF). The femtosecond laser pulse of around 400 nm (SH, second harmonic) is selected. (**b**), Example patterns for dual encoding from two reflection directions of DMD. C_1 and C_2 are complementary. (**c**), Illustration of two polarizers in two complementarily encoded beam paths. P1 and P2 have polarization axes in the vertical (y) and horizontal (x) directions, respectively. (**d**), The section of the system where the femtosecond laser pulse of around 800 nm (fundamental) is selected to excite two-photon fluorescence (2PF). The sections in dashed boxes are interchangeable for either 1PF or 2PF.

Table S1: Detail information of the components used in CUP2AI.

Component	Abbreviation	Manufacturer	Model number
Femtosecond laser	--	Coherent	Libra HE
Ultrafast mirror	M	Thorlabs	UM10-AG
Nonlinear crystal (beta barium borate)	BBO	United Crystals	Custom-made
Short-pass filter (cut-off @ 450 nm)	SPF	Omega Filters	RPE450-SP
Long-pass filter (cut-off @ 550 nm)	LPF	Thorlabs	FEL0550
Cylindrical lens (f = 500 mm)	CyL	Thorlabs	LJ1144RM
Half-wave plate (achromatic)	HWP	Thorlabs	AHWP10M-580
Polarizer (broadband)	P0	Thorlabs	GL15
Beamsplitter (10:90, R:T)	BS0	Thorlabs	BS040
Beam dump	BD	Thorlabs	BT610
Power meter	PM	Thorlabs	PM100USB
Lens (f = 200 mm)	L1 [a]	Thorlabs	AC508-200-A
Lens (f = 100 mm)	L2 [b]	Thorlabs	AC508-100-A
Band-pass filter (λ_c = 520 nm, BW = 70 nm)	BPF (1) [c]	Semrock	FF01-520/70-25
Band-pass filter (λ_c = 460 nm, BW = 60 nm)	BPF (2) [c]	Semrock	FF01-460/60-25
Beamsplitter (50:50, R:T)	BS	Thorlabs	BS013
External CCD camera	--	Point Gray	CM3-U3-28S4M-CS
Relay lens (f = 150 mm)	ReL0	Thorlabs	AC508-150-A
Stereoscopic lens	SL	Olympus	MV PLAPO 2XC
Digital micro-mirror device	DMD	Texas Instruments	LightCrafter 3000
Polarizer	P1 & P2	Thorlabs	LPVISE100-A
Relay lens (f = 200 mm)	ReL1 & ReL2	Thorlabs	AC254-200-A
Knife-edge prism mirror	KRPM	OptoSigma	KRPB4-15-550
Streak camera	--	Hamamatsu	C7700

Notes:

[a] L1 is part of ImL, as marked in Fig. S1a and Fig. 1a.

[b] L2 is part of ImL, as marked in Fig. S1a and Fig. 1a.

[c] These two options of BPFs are marked as BPF in Fig. 1a.

2. Generation and characterization of laser sheets

The original 800-nm fs-pulse is linearly polarized in the y-direction, while the 400-nm SH has linear polarization along the x-direction. A broadband half-wave plate (HWP) and a polarizer (P0) together make sure that the laser sheet is always linearly polarized along the y-direction (blue arrow in Fig. 1a and green arrows in Figs. S2a and S2d). We can also continuously tune the laser fluence by rotating the HWP.

A camera, placed at the focus of the cylindrical lens (CyL), measures the 2D profiles of the laser sheets. The results are shown in Fig. S2, in which the normalized intensities along y and z directions are given as well. The coordinates are defined in Fig. S1. Table S2 summarizes the measured parameters of laser sheets used in different experiments.

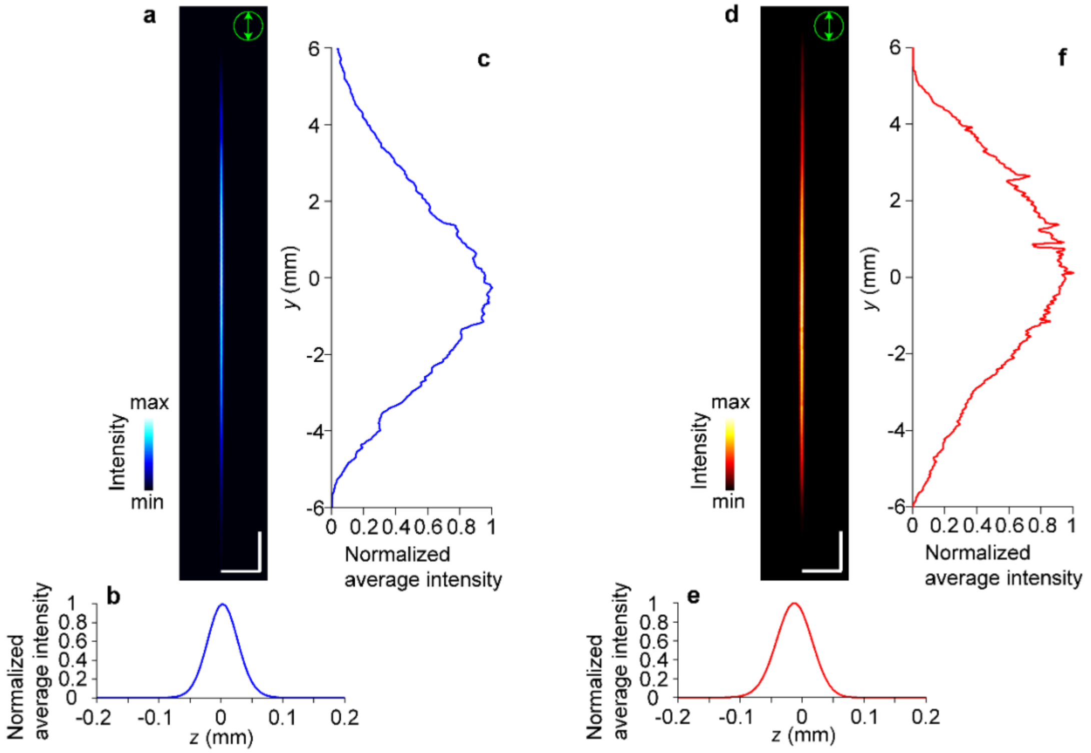

Fig. S2. Intensity profiles of laser sheets in the y-z coordinates, measured by a camera at the focus of the cylindrical lens, where the samples are placed. The definitions of y and z directions are in Fig. S1a. (**a**) – (**c**), Laser sheet for one-photon fluorescence. (**d**) – (**f**), Laser sheet for two-photon fluorescence. (**a**) and (**d**), 2D intensity profiles. Their directions of polarization (y) are labeled. Scale bars: 1 mm. (**b**) and (**e**), average intensity profiles along the z direction. (**c**) and (**f**), average intensity profiles along the y direction. Plots in (**b**), (**c**), (**e**), and (**f**) are normalized.

Table S2: Parameters of laser sheets.

Experiment	Figures & Movies	Laser pulse	Laser sheet size ($y \times z$)	Power	Rep. rate	Fluence of a single pulse
1PF in liquid (fluorescein, 4K, and 20K molecules)	Fig. 2 & Movies S1 – S5	400-nm (SH)	0.058 mm × 5.50 mm	5 mW	500 Hz	3.1 mJ cm^{-2}
1PF in gas (PAH in flame)	Fig. 4 & Movies S6, S7	400-nm (SH)	0.058 mm × 5.50 mm	40 mW	500 Hz	25 mJ cm^{-2}
2PF in liquid (fluorescein, 4K, and 20K molecules)	Fig. 6 & Movie S8	800-nm	0.066 mm × 5.73 mm	257 mW	500 Hz	136 mJ cm^{-2}

To demonstrate 2PF excitation, we imaged the laser sheet profiles inside the fluorescein sample in the x-y plane. The full-width-at-half-maximum (FWHM) of the laser sheet for 2PF in the y-direction is $\sqrt{2}$ times smaller than that of the laser sheet for 1PF, which is typical for 2PF.

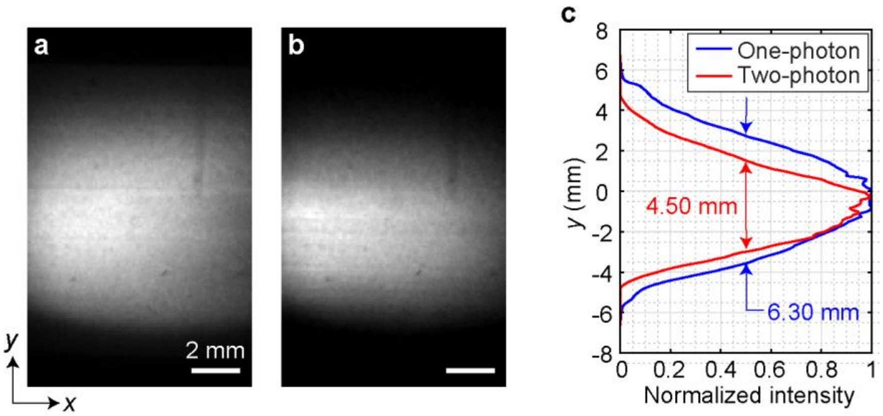

Fig. S3. Intensity profiles (in the x-y plane) for the laser sheets in fluorescein when no pattern masks are applied on the cuvette. These are imaged by a traditional camera (external CCD camera in Fig. S1). The laser pulses have the same fluences used in CUP2AI imaging. Proper ND filters are used to avoid saturation. (**a**) One-photon fluorescence (1PF). (**b**) Two-photon fluorescence (2PF). (**c**) Normalized intensity profiles along y direction. The FWHMs in the y direction of the laser sheets used in 1PF and 2PF are 6.30 mm and 4.50 mm, respectively. Scale bars in (**a**) and (**b**): 2 mm.

3. Laser spectra and fluorescence filters

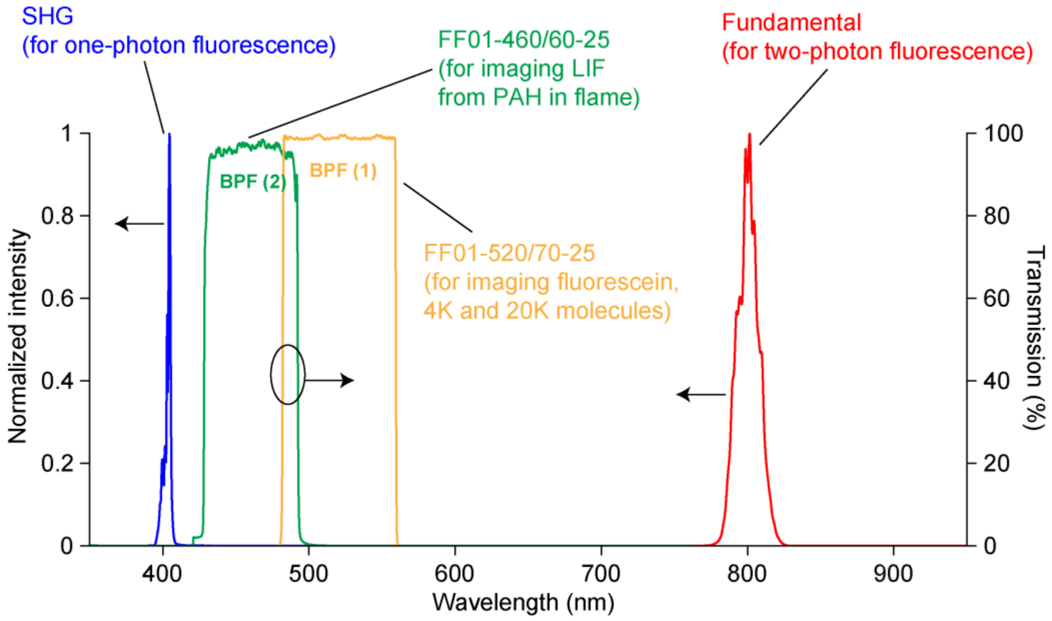

Fig. S4. Spectrum measurements of the laser pulses and spectral filters used in the CUP2AI system. Blue line: 400-nm femtosecond pulse for one-photon fluorescence (1PF) excitation. Red line: 800-nm femtosecond pulse for two-photon fluorescence (2PF) excitation. Green line: transmission of the bandpass filter used for imaging 1PF from PAH molecules in gaseous environment (flame). Orange line: transmission of the bandpass filter used for imaging 1PF and 2PF from fluorescein, 4K, and 20K molecules in liquid environment. The circles and arrows group the plots for either left-y axis or right-y axis.

4. Streak camera

The structure of a typical streak camera is illustrated in Fig. S5a, where an ultrafast sweeping voltage is applied to the electrodes, deflecting photoelectrons in the vertical (y_s) direction. The coordinates (x_s-y_s-z_s) of the streak camera are defined, and photoelectrons arriving at different times (t_1), $e(t_2)$ and $e(t_3)$ are shown. In Fig. S5b, the image formation process of CUP2AI is depicted, where image pairs encoded by C_1 and C_2 are captured side by side on the photocathode, and the dual images on the CMOS sensor undergo temporal shearing and integration. The direction of shearing and displacements of representative frames are indicated. Note that the exact relation between the applied sweeping voltage and electron displacement is determined by the streak camera manufacturer (Hamamatsu) and remains inaccessible. However, a qualitative representation is provided in the inset of Fig. S5a. The system allows for selecting different pre-set sweeping speeds, with a general rule that a higher sweeping voltage results in a greater displacement between electrons arriving at different times, thereby increasing the imaging speed for CUP2AI.

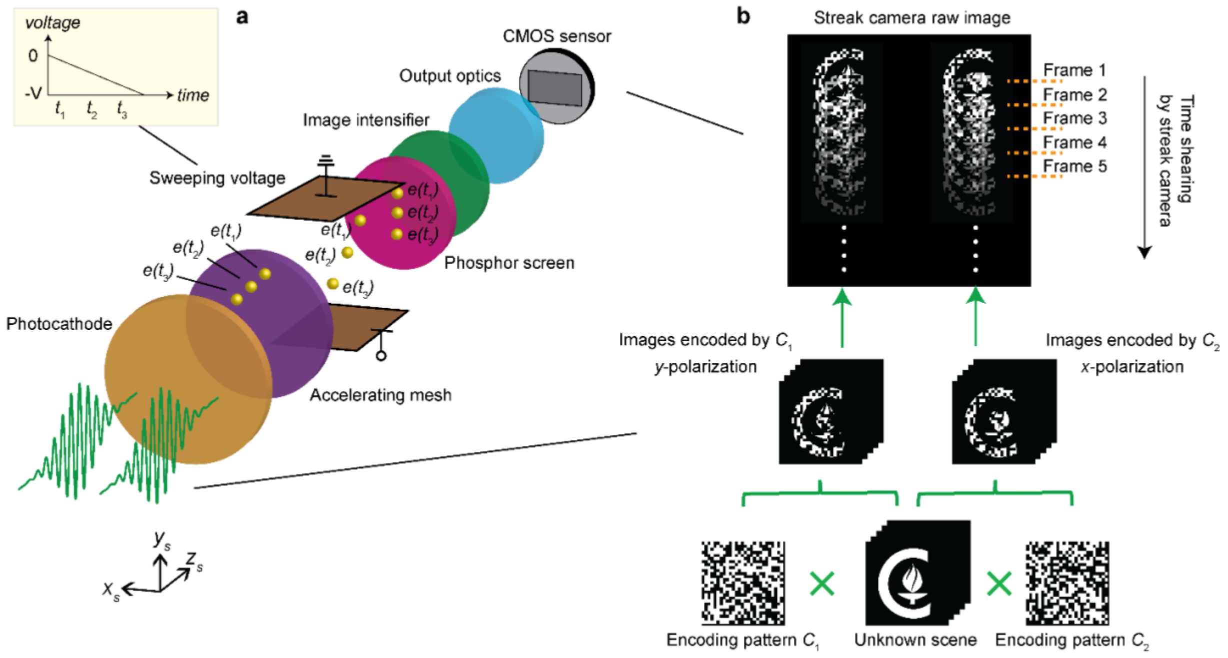

Fig. S5. Streak camera and CUP2AI's image formation process. (**a**), The detailed structure inside a typical streak camera. An ultrafast sweeping voltage is applied on the electrodes to deflect photoelectrons in the vertical (y_s) direction. Here, $e(t_1)$, $e(t_2)$ and $e(t_3)$ represent photoelectrons coming at different times. The coordinates of the streak camera (x_s-y_s-z_s) are defined. (**b**), Illustration of image formation process of CUP2AI. The pair of images encoded by C_1 and C_2 are captured by the photocathode side by side. The dual images on the CMOS sensor are temporally sheared and integrated. The direction of temporal shearing and the displacements of five representative frames are shown.

5. Temporal response and deconvolution

5.1. *Temporal resolution*

The temporal resolutions at different imaging speeds are characterized by imaging a binary pattern illuminated by a single femtosecond laser pulse. Since this simple ultrafast event is repeatable and stable, the results from multiple trials are similar. Therefore, we did not average over multiple shots for this calibration. The full-width-at-half-maximum (FWHM) of the reconstructed temporal profile is defined as the temporal resolution [1, 2]. The numerically fit curves are shown in Fig. S6. The root-mean-square (RMS) of the fitting error is less than 2% for all cases. The temporal resolutions for different experiments are summarized in Table S3.

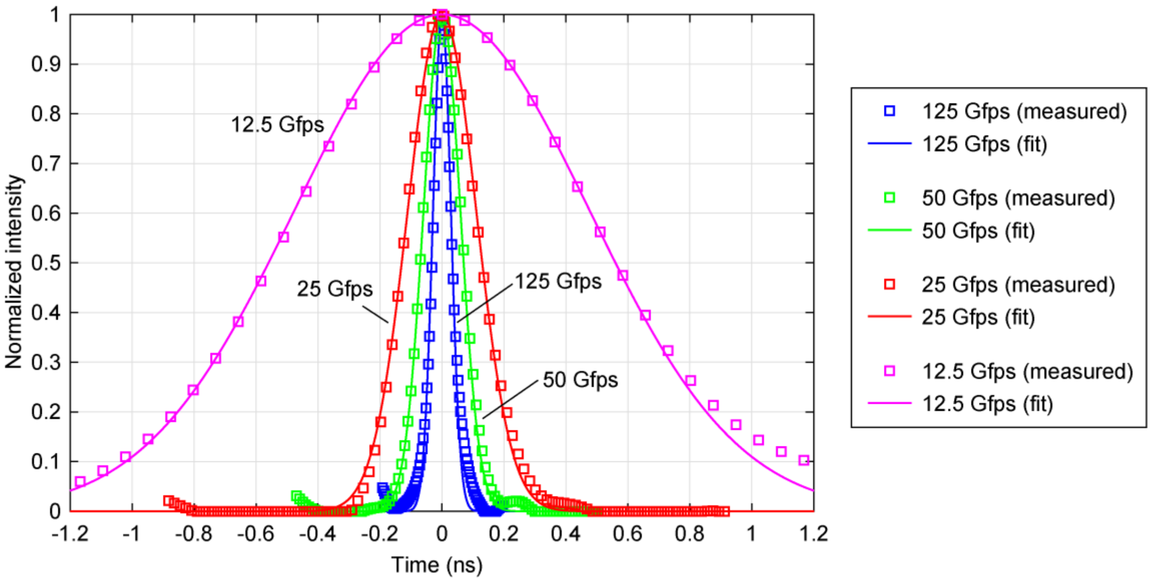

Fig. S6. CUP2AI's temporal point-spread-functions (PSFs). Blue, green, red, and magenta lines are PSFs at 125-Gfps, 50-Gfps, 25-Gfps, and 12.5-Gfps imaging speeds. They are fits to Gaussian functions. The experimental measurements are plotted as squares in the same colors.

Table S3: Imaging speeds and temporal resolutions of different CUP2AI configurations.

Experiment	Figures & Movies	Imaging speed	Frame interval	Temporal resolution
1PF in liquid (fluorescein, 4K, and 20K molecules)	Figs. 2 & 3, Movies S1 – S5	125 Gfps	8 ps	0.068 ns

1PF in gas (PAH in flame)	Figs. 5a – 5h, Movie S6	50 Gfps	20 ps	0.13 ns
1PF in gas (PAH in flame)	Figs. 4 & 5i – 5p, Movie S7	25 Gfps	40 ps	0.24 ns
2PF in liquid (fluorescein, 4K, and 20K molecules)	Fig. 6, Movie S8	12.5 Gfps	80 ps	1.12 ns

5.2 *Temporal convolution and deconvolution*

Two examples of temporal convolution of single-exponential decay curves with calibrated CUP2AI temporal PSFs are shown in Fig. S7, demonstrating how the decay time is lengthened by the system's finite temporal resolution. To estimate the original decay time based on the measured decay time (after convolution) and the calibrated temporal PSF, we built a look-up-table (LUT) via numerical calculations over a wide range of decay times. This LUT is plotted in Fig. S8, where it is straightforward to identify the original decay time (horizontal axis) from the measured decay time (vertical axis) for a specific imaging speed. This is used as a method to approximate an actual deconvolution process and is implemented point-by-point in all anisotropy lifetime maps shown in Figs. 2e, 2i, 2m, 2q, 2u, 4f, 4i, 4m, 4p, 6d, 6g, 6j, 6m, S10d, S10j, S11d, S11j, S12d, S12j, S13d, S13j, S14d, S14j, S15d, S15g, S16d, and S16g.

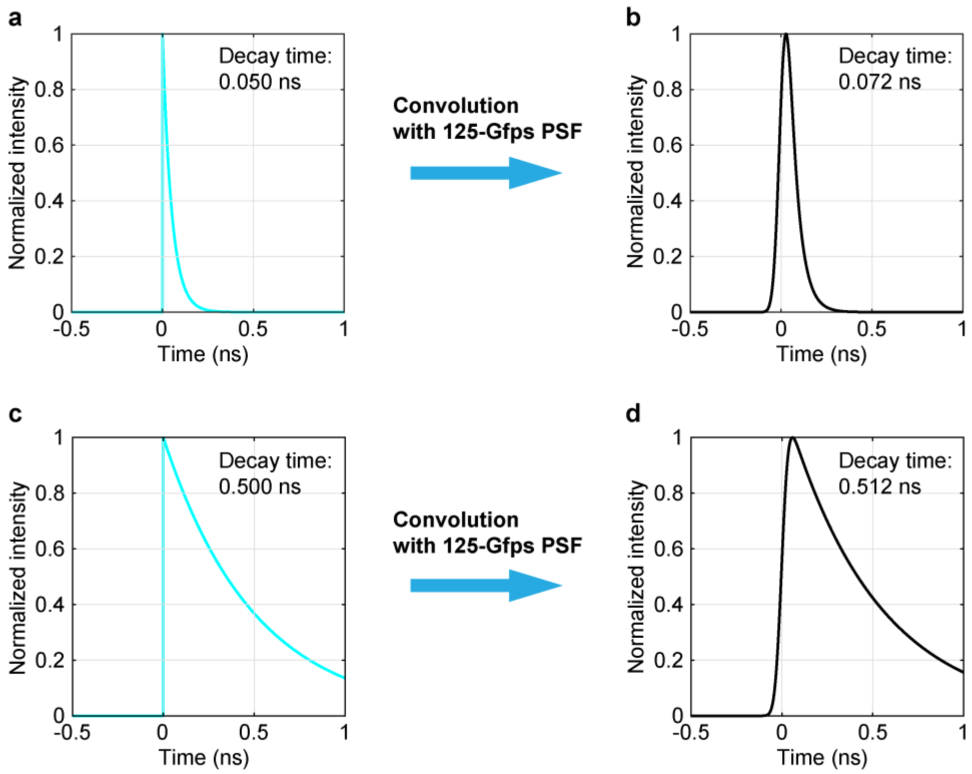

Fig. S7. Two examples of temporal convolution of the ground truth intensity evolution (exponential decay) with the temporal PSFs of CUP2AI. The imaging speed is assumed to be 125 Gfps. These are numerical calculations. (**a**) and (**b**), first example with an original temporal decay time of 0.050 ns. (**c**) and (**d**), second example with an original temporal decay time of 0.500 ns. (**a**) and (**c**), original signals. (**b**) and (**d**), after convolution. The final decay times are 0.072 ns and 0.512 ns, respectively.

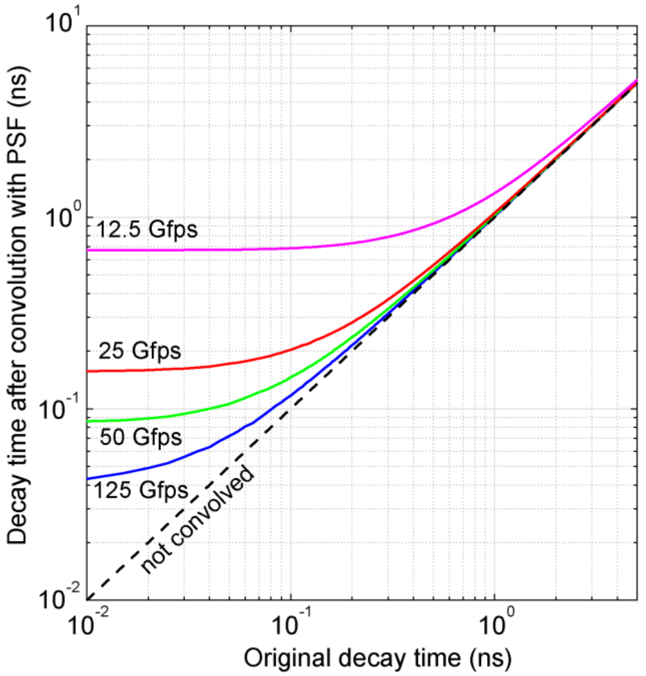

Fig. S8. Quasi-deconvolution calibration curves of CUP2AI at different imaging speeds. The horizontal axis represents the original decay time, while the vertical axis is the decay time after convolution with the corresponding temporal PSFs. The black dashed line is the unconvolved decay constant, used to manifest the impact of the approach approximating a deconvolution.

It is worth noting that for short decay times (e.g. < 100 ps), the deconvolution calibration curve (in Fig. S8) of a slower imaging speed is flatter (smaller slope angle) than that of a faster speed. This results in poorer accuracy in numerical deconvolution when the imaging speed is slower. This explains the compromise in spatial variations of molecule sizes using 2PF (see Figs. S19c, S19d) compared to those using 1PF (see Figs, 3c, 3d).

6. Data processing overview

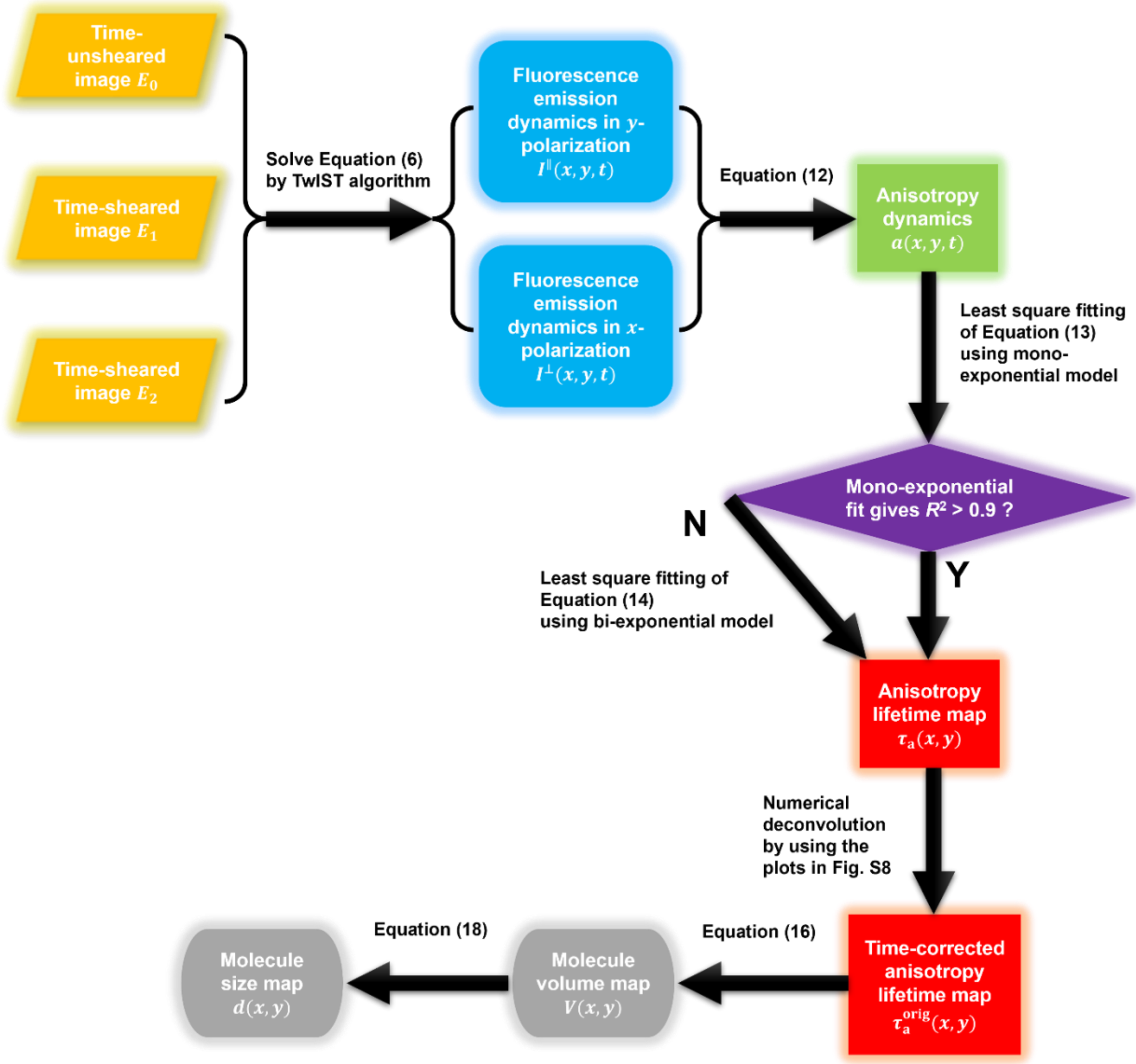

Fig. S9. Flow chart of the entire data processing streamline in CUP2AI. The operations connecting data blocks are given as well.

7. **<u>Fluorescence sample in liquid</u>**

Table S4: Parameters of fluorescent samples in liquid environment.

Fluorescent molecule	Molar mass	Powder weight	Water	Molar Concentration	Vendor's webpage
Fluorescein	332.3 g/mol	0.165 mg	10 mL	50 µM	https://www.sigmaaldrich.com/US/en/product/sigma/46955
FITC-dextran 4K	4,000 g/mol	2 mg	10 mL	50 µM	https://www.sigmaaldrich.com/US/en/product/sigma/46944
FITC-dextran 20K	20,000 g/mol	10 mg	10 mL	50 µM	https://www.sigmaaldrich.com/US/en/product/sigma/fd20

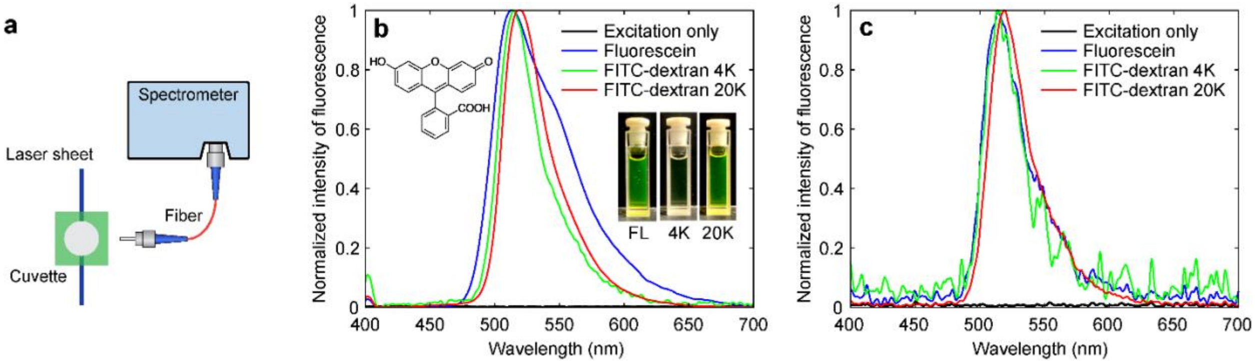

Fig. S10. Spectrum measurements of fluorescent molecules in liquid environment. (**a**) Schematic of measurement setup, in which a fiber spectrometer (Ocean Optics) is placed close to the cuvette. (**b**) One-photon fluorescence (1PF) emission spectra of fluorescein, FITC-dextran 4K, and FITC-dextran 20K molecules excited by the 400-nm laser pulse. Left inset: molecular structure of fluorescein. Right inset: photographs of the three samples in cuvettes. (**c**) Two-photon fluorescence (2PF) emission spectra of fluorescein, FITC-dextran 4K, and FITC-dextran 20K molecules excited by the 800-nm laser pulse. Note that black lines are background measurements taken with excitation laser only and without any sample.

8. Additional results of one-photon fluorescence (1PF) in liquid environment

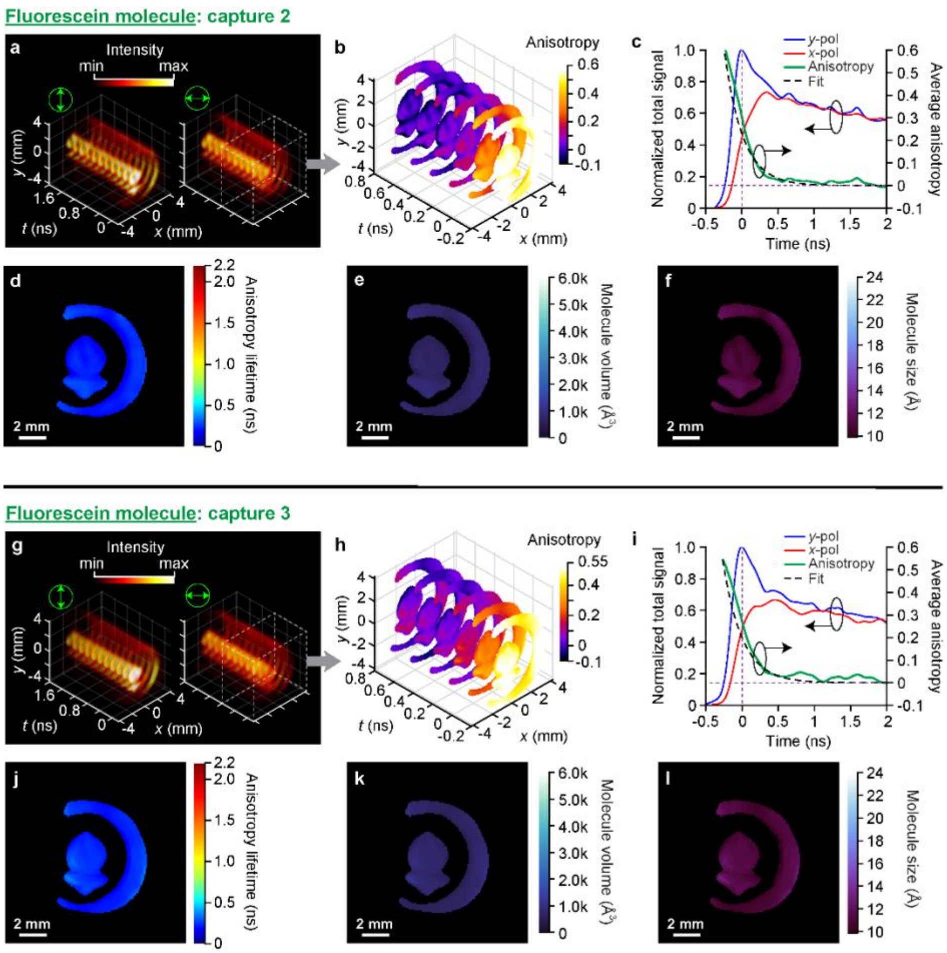

Fig. S11. Additional results of 1PF of fluorescein, imaged at 125-Gfps. The first acquisition is in Figs. 2 and 3. (**a**) – (**f**), the second acquisition. (**g**) – (**l**), the third acquisition. (**a**) and (**g**), Reconstructed intensity evolutions. 12 exemplary snapshots are selected. Left panels: y-polarization. Right panels: x-polarization. The intensity is normalized to the maximum signal between the two channels. (**b**) and (**h**), Polarization anisotropy evolutions, over the first 1.0 ns. 6 exemplary snapshots are shown for clarity. (**c**) and (**i**), Left y-axis: evolutions of normalized spatially integrated intensities from both polarization channels. Right y-axis: evolutions of spatially averaged anisotropy. (**d**) and (**j**), Anisotropy lifetime maps. (**e**) and (**k**), Maps of molecule volumes. (**f**) and (**l**), Maps of molecule size. The circles and arrows in (**c**) and (**i**) group the plots for either left-y axis or right-y axis.

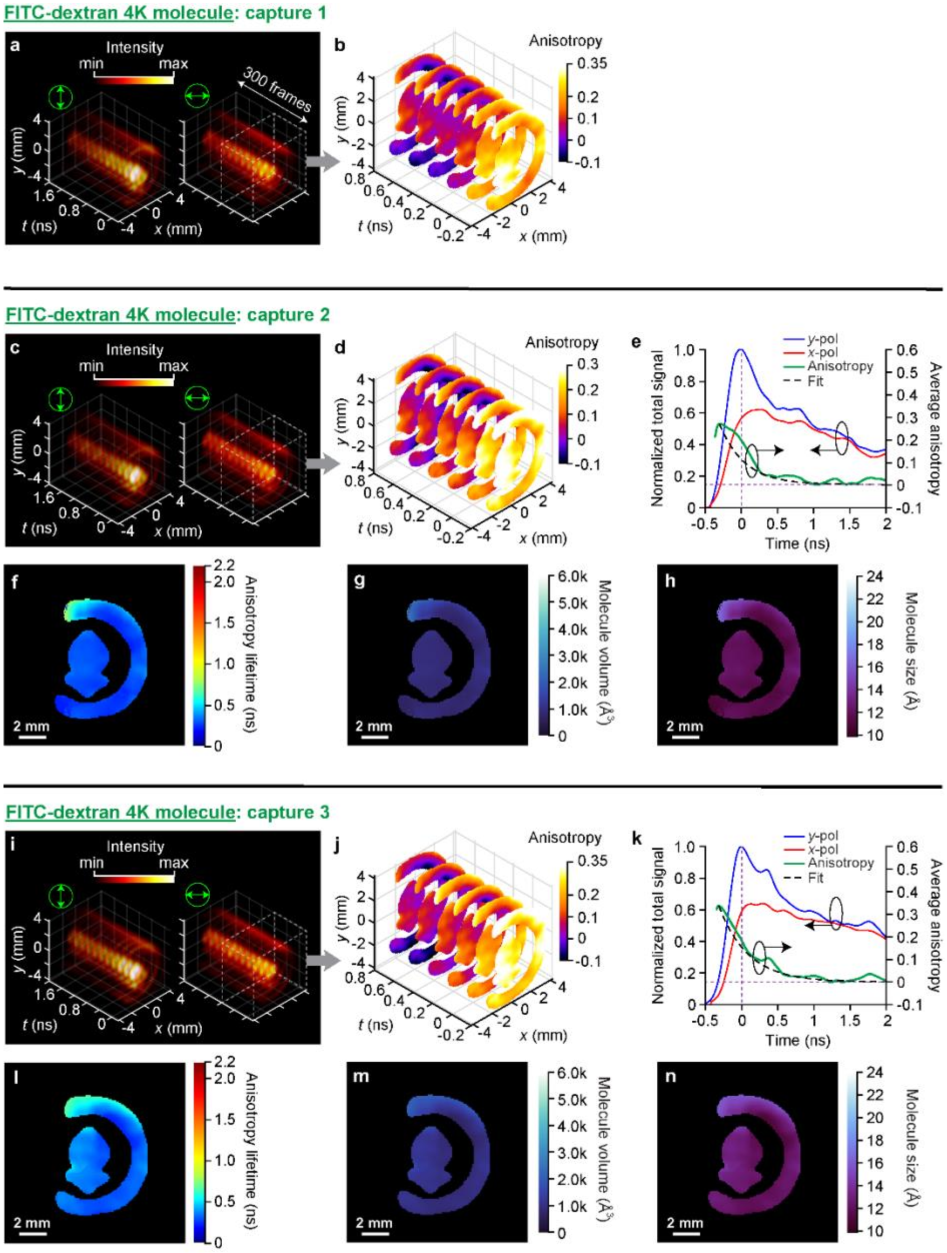

Fig. S12. Additional results of 1PF of FITC-dextran 4K molecule, imaged at 125-Gfps. (**a**) and (**b**), the first acquisition, the rest of results are in Figs. 2 and 3. (**c**) – (**h**), the second acquisition. (**i**) – (**n**), the third acquisition. (**a**), (**c**), and (**i**), Reconstructed intensity evolutions. 12 exemplary snapshots are selected. Left panels: y-polarization. Right panels: x-polarization. The intensity is normalized to the maximum signal between the two channels. (**b**), (**d**), and (**j**), Polarization anisotropy evolutions, over the first 1.0 ns. 6 exemplary snapshots are shown for clarity. (**e**) and (**k**), Left y-axis: evolutions of normalized spatially integrated intensities from both polarization channels. Right y-axis: evolutions of spatially averaged anisotropy. (**f**) and (**l**), Anisotropy lifetime maps. (**g**) and (**m**), Maps of molecule volumes. (**h**) and (**n**), Maps of molecule size. The circles and arrows in (**e**) and (**k**) group the plots for either left-y axis or right-y axis.

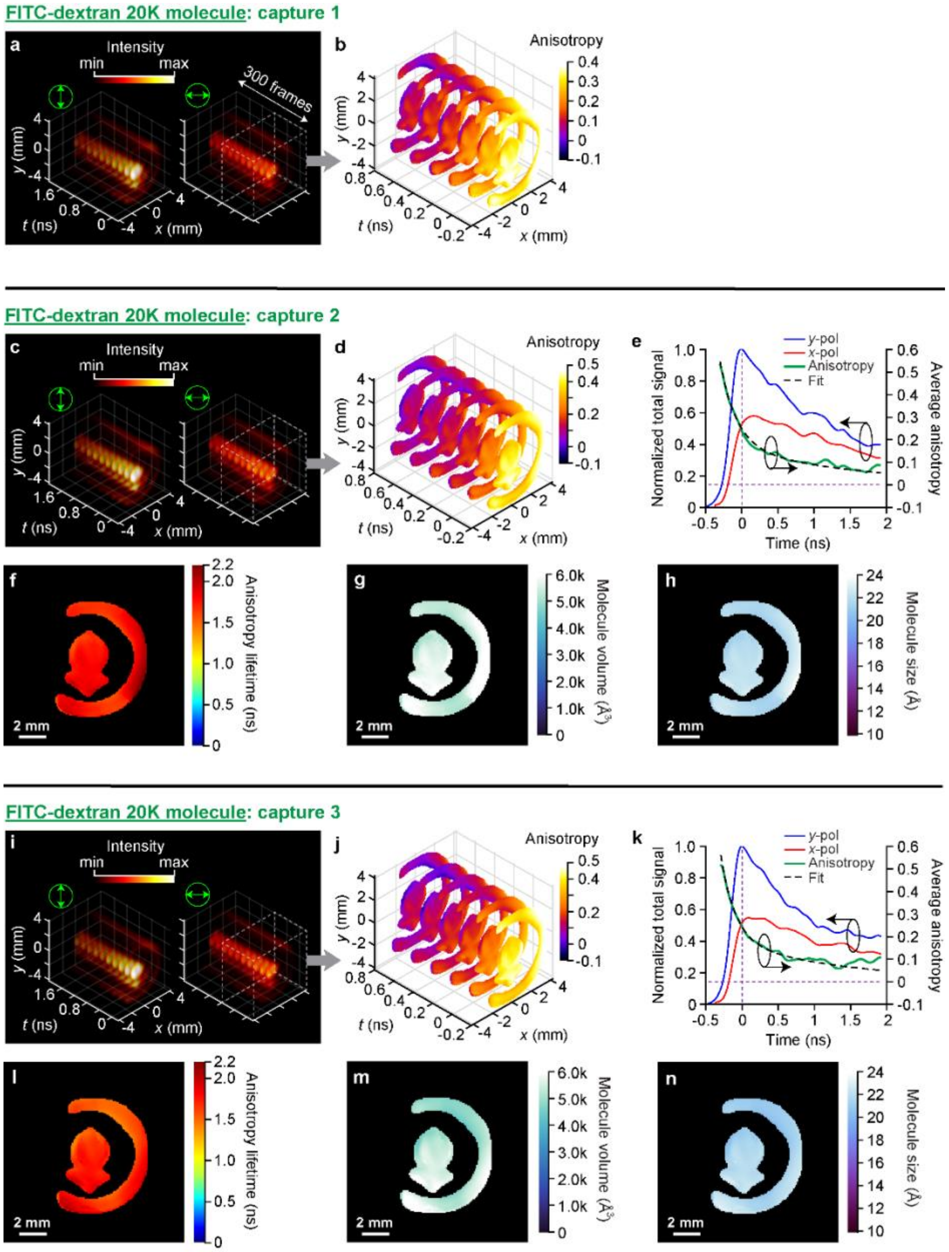

Fig. S13. Additional results of 1PF of FITC-dextran 20K molecule, imaged at 125-Gfps. (**a**) and (**b**), the first acquisition, the rest of results are in Figs. 2 and 3. (**c**) – (**h**), the second acquisition. (**i**) – (**n**), the third acquisition. (**a**), (**c**), and (**i**), Reconstructed intensity evolutions. 12 exemplary snapshots are selected. Left panels: y-polarization. Right panels: x-polarization. The intensity is normalized to the maximum signal between the two channels. (**b**), (**d**), and (**j**), Polarization anisotropy evolutions, over the first 1.0 ns. 6 exemplary snapshots are shown for clarity. (**e**) and (**k**), Left y-axis: evolutions of normalized spatially integrated intensities from both polarization channels. Right y-axis: evolutions of spatially averaged anisotropy. (**f**) and (**l**), Anisotropy lifetime maps. (**g**) and (**m**), Maps of molecule volumes. (**h**) and (**n**), Maps of molecule size. The circles and arrows in (**e**) and (**k**) group the plots for either left-y axis or right-y axis.

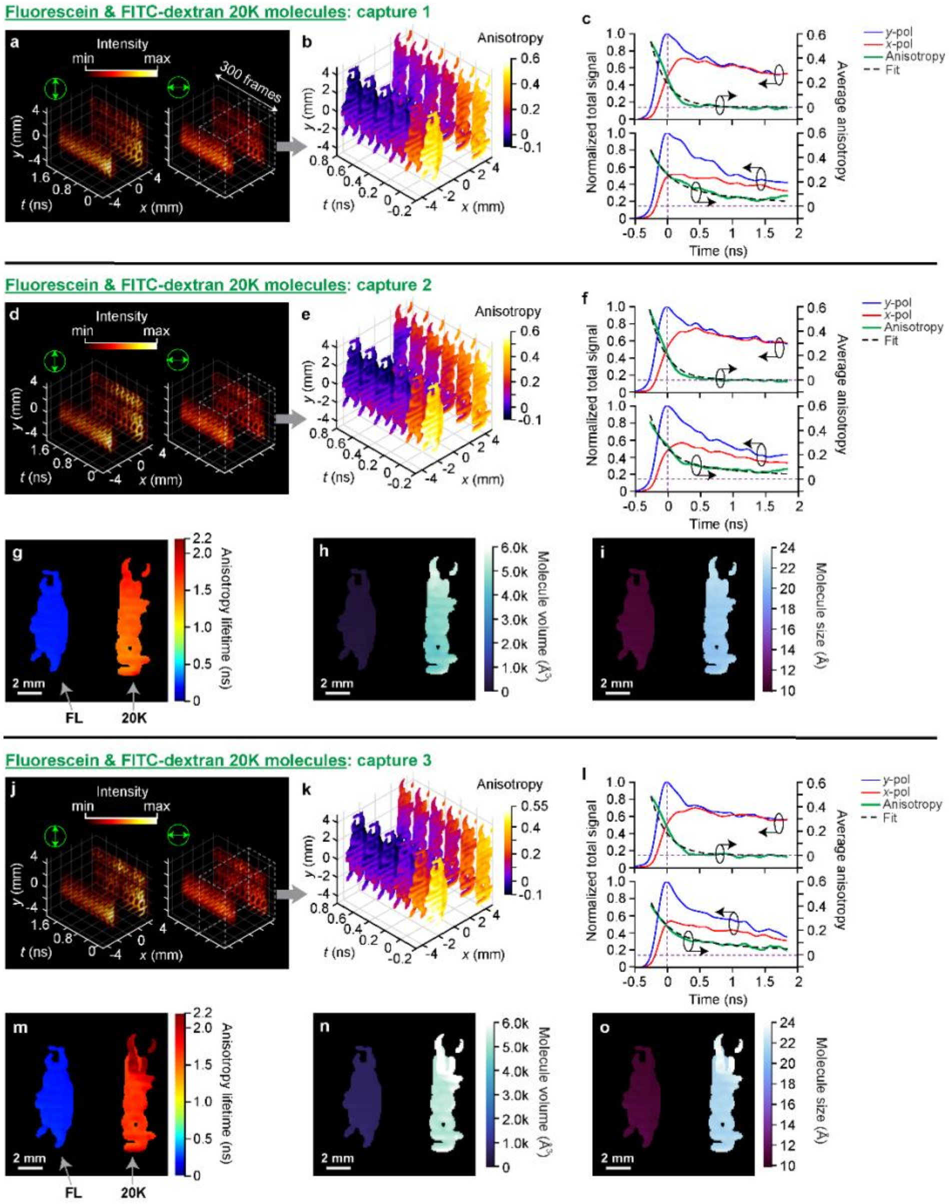

Fig. S14. Additional results of 1PF of fluorescein and FITC-dextran 20K molecules, imaged at 125-Gfps. (**a**) – (**c**), the first acquisition, the rest of results are in Figs. 2 and 3. (**d**) – (**i**), the second acquisition. (**j**) – (**o**), the third acquisition. (**a**), (**d**), and (**j**), Reconstructed intensity evolutions. 12 exemplary snapshots are selected. Left panels: y-polarization. Right panels: x-polarization. The intensity is normalized to the maximum signal between the two channels. (**b**), (**e**), and (**k**), Polarization anisotropy evolutions, over the first 1.0 ns. 6 exemplary snapshots are shown for clarity. (**c**), (**f**), and (**l**), Left y-axis: evolutions of normalized spatially integrated intensities from both polarization channels. Right y-axis: evolutions of spatially averaged anisotropy. Top plots are for fluorescein and bottom plots are for 20K molecule. (**g**) and (**m**), Anisotropy lifetime maps. (**h**) and (**n**), Maps of molecule volumes. (**i**) and (**o**), Maps of molecule size. The circles and arrows in (**c**), (**f**), and (**l**) group the plots for either left-y axis or right-y axis.

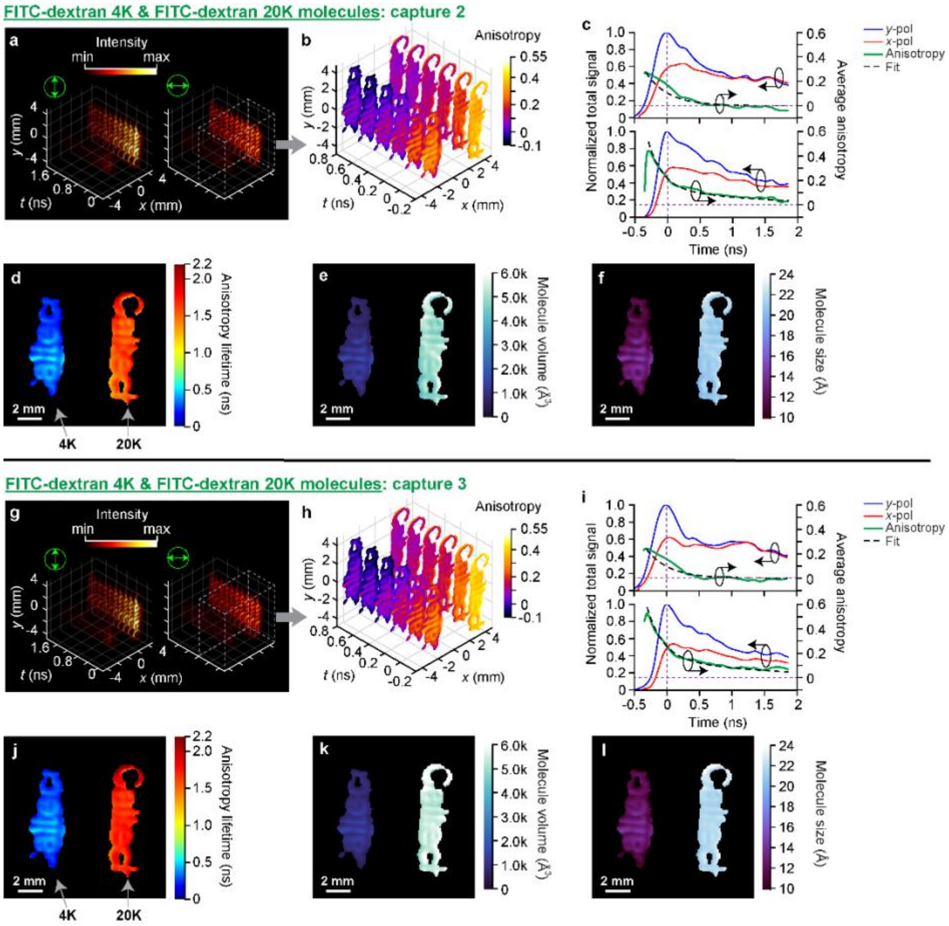

Fig. S15. Additional results of 1PF of FITC-dextran 4K and FITC-dextran 20K molecules, imaged at 125-Gfps. The first acquisition is in Figs. 2 and 3. (**a**) – (**f**), the second acquisition. (**g**) – (**l**), the third acquisition. (**a**) and (**g**), Reconstructed intensity evolutions. 12 exemplary snapshots are selected. Left panels: y-polarization. Right panels: x-polarization. The intensity is normalized to the maximum signal between the two channels. (**b**) and (**h**), Polarization anisotropy evolutions, over the first 1.0 ns. 6 exemplary snapshots are shown for clarity. (**c**) and (**i**), Left y-axis: evolutions of normalized spatially integrated intensities from both polarization channels. Right y-axis: evolutions of spatially averaged anisotropy. Top plots are for 4K molecule and bottom plots are for 20K molecule. (**d**) and (**j**), Anisotropy lifetime maps. (**e**) and (**k**), Maps of molecule volumes. (**f**) and (**l**), Maps of molecule size. The circles and arrows in (**c**) and (**i**) group the plots for either left-y axis or right-y axis.

9. Additional results of one-photon fluorescence (1PF) in gaseous environment

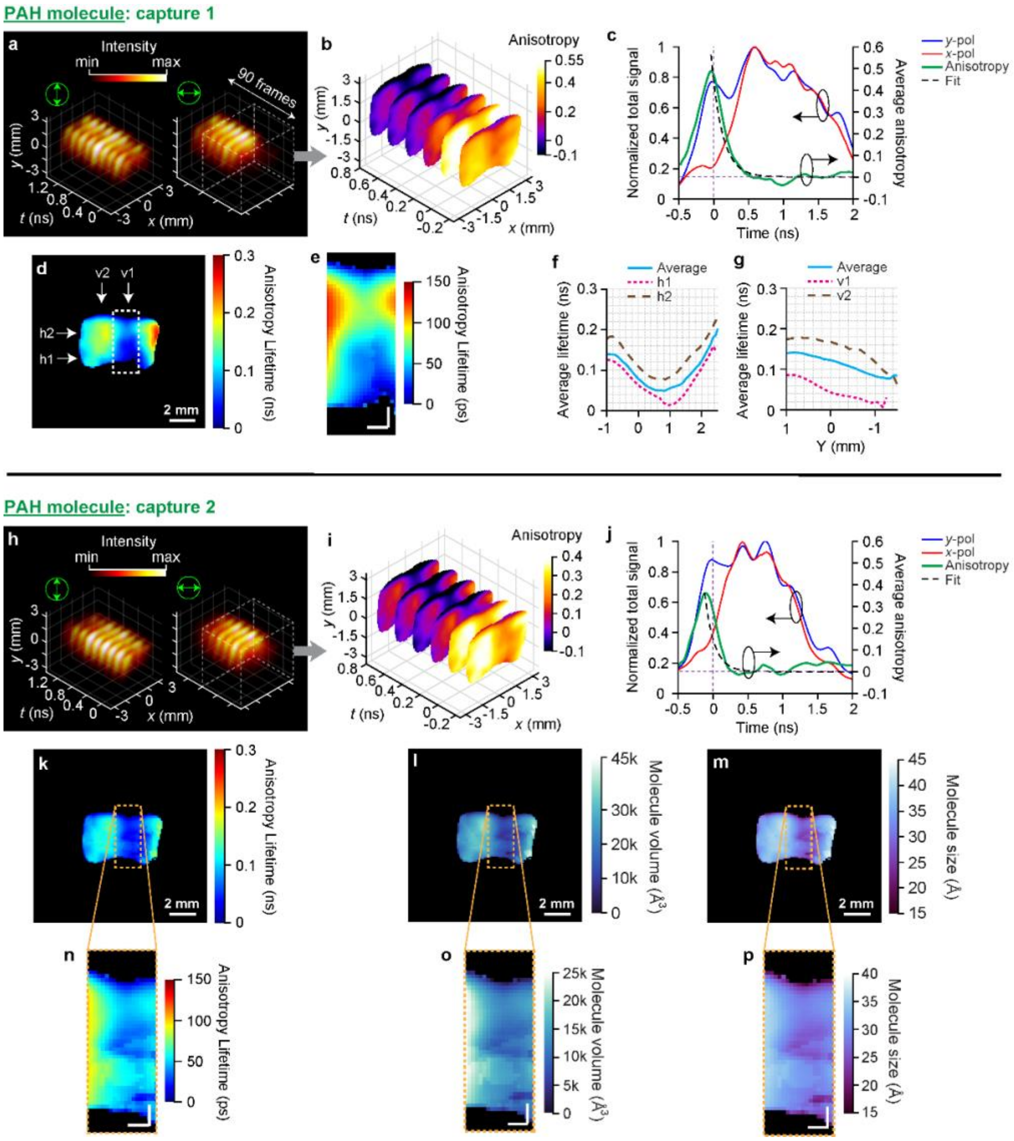

Fig. S16. Additional results of 1PF of PAH molecules in flame, imaged at 50-Gfps. (**a**) – (**g**), the first acquisition, the rest of results are in Figs. 4 and 5. (**h**) – (**p**), the second acquisition. (**a**) and (**h**), Reconstructed intensity evolutions. 9 exemplary snapshots are selected among a total of 128 frames. Left panels: y-polarization channel. Right panels: x-polarization channel. The intensity is normalized to the maximum signal between the two channels. (**b**) and (**i**), Polarization anisotropy evolutions, over the first 1.0 ns. 6 exemplary snapshots are shown for clarity. (**c**) and (**j**), Left y-axis: evolutions of normalized spatially integrated intensities from both polarization channels. Right y-axis: evolutions of spatially averaged anisotropy. (**d**) and (**k**), Anisotropy lifetime maps. (**e**) and (**n**), Magnified views of anisotropy lifetimes in (**d**) and (**k**). (**f**) and (**g**), Average anisotropy lifetimes along (**f**) the x-direction [h1 and h2 labeled in (**d**)] and (**g**) the y-direction [v1 and v2 labeled in (**d**)]. (**l**), Molecule volume map. (**m**), Molecule size map. (**o**) and (**p**), Magnified views of (**o**) molecule volumes and (**p**) molecule sizes in the center region. The colormaps in (**e**), (**n**), (**o**), and (**p**) are rescaled. The circles and arrows in (**c**) and (**j**) group the plots for either left-y axis or right-y axis.

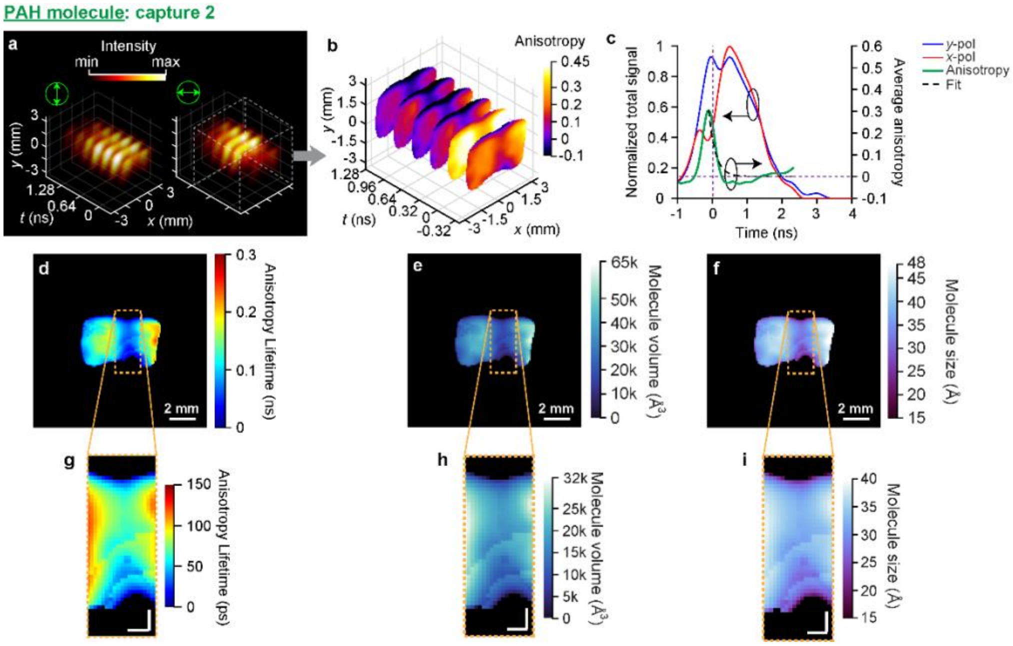

Fig. S17. Additional results of 1PF of PAH molecules in flame, imaged at 25-Gfps. The first acquisition is in Figs. 4 and 5. These are the second acquisition. (**a**), Reconstructed intensity evolutions. 7 exemplary snapshots are selected among a total of 103 frames. Left panels: y-polarization channel. Right panels: x-polarization channel. The intensity is normalized to the maximum signal between the two channels. (**b**), Polarization anisotropy evolutions, over the first 1.6 ns. 6 exemplary snapshots are shown for clarity. (**c**), Left y-axis: evolutions of normalized spatially integrated intensities from both polarization channels. Right y-axis: evolutions of spatially averaged anisotropy. (**d**), Anisotropy lifetime map. (**e**), Molecule volume map. (**f**), Molecule size map. (**g**) – (**i**), Magnified views of (**g**) anisotropy lifetimes, (**h**) molecule volumes, and (**i**) molecule sizes in the center regions of the flame, enclosed by the dashed boxes in (**d**), (**e**), and (**f**). The colormaps in (**g**), (**h**), and (**i**) are rescaled. The circles and arrows in (**c**) group the plots for either left-y axis or right-y axis.

10. Additional results of two-photon fluorescence (2PF) in liquid environment

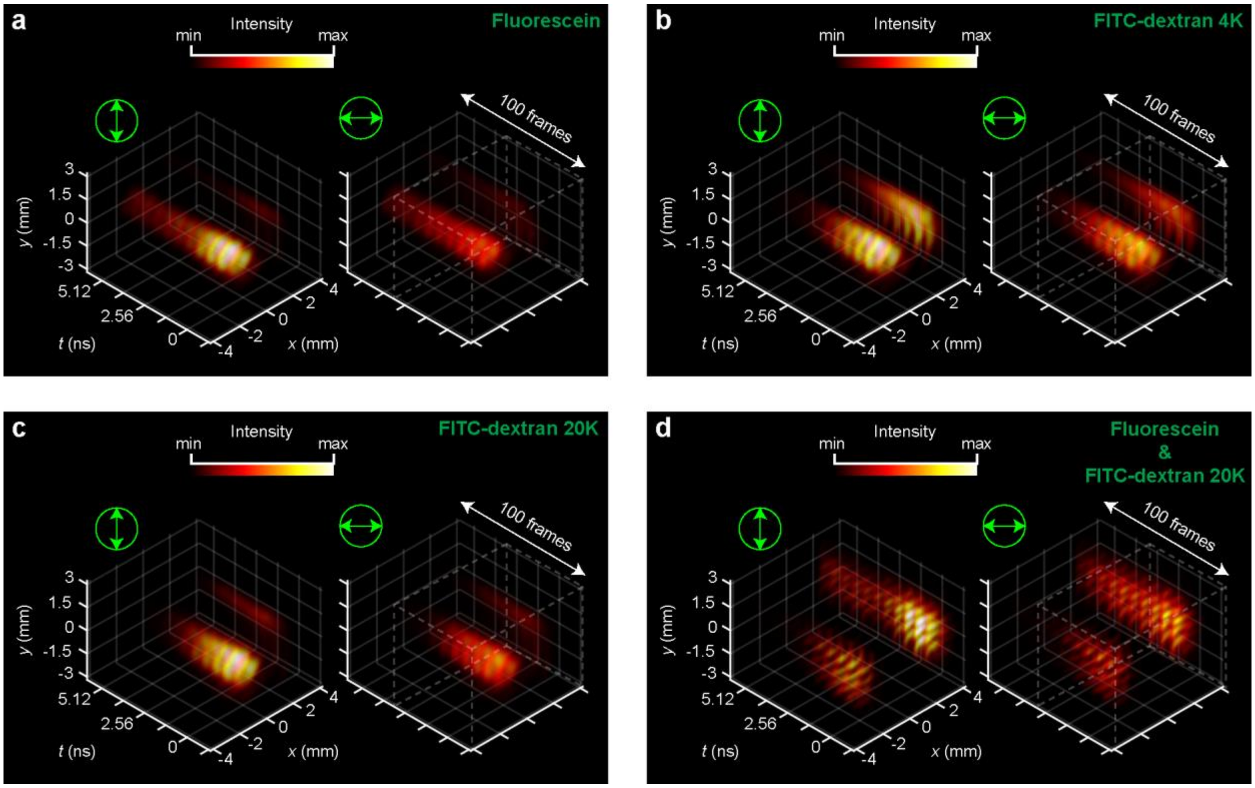

Fig. S18. Reconstructed intensity evolutions of 2PF in liquid environment, imaged by CUP2AI at 12.5-Gfps. 11 exemplary snapshots over 7.4 ns are selected among a total of 100 frames. Left panels: y-polarization channel. Right panels: x-polarization channel. The intensity is normalized to the maximum signal between the two channels. (**a**) Fluorescein. (**b**) FITC-dextran 4K molecule. (**c**) FITC-dextran 20K molecule (**d**) Both fluorescein and FITC-dextran 20K molecules. The green double-headed arrows represent the orthogonal orientations of light polarization.

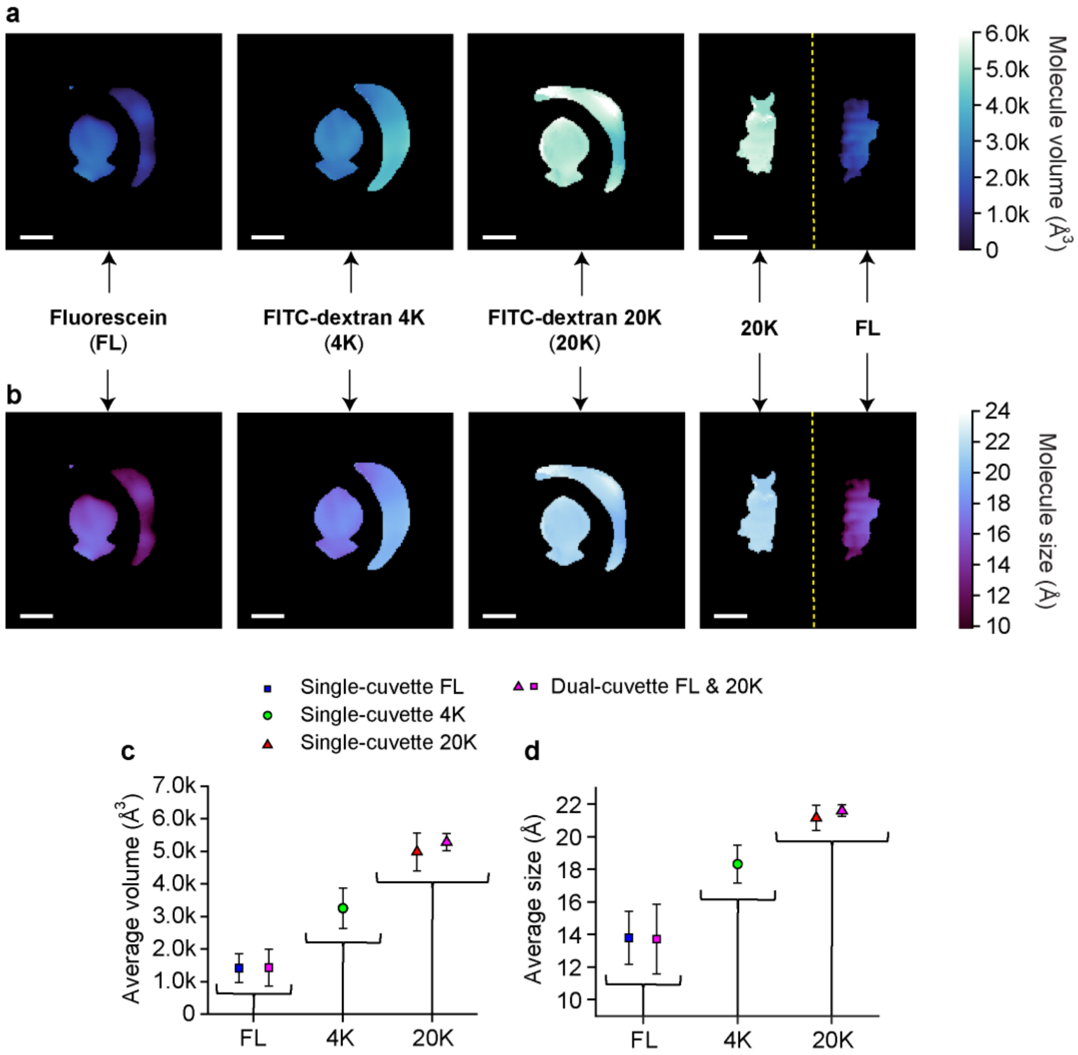

Fig. S19. Molecule size maps based on the 12.5-Gfps 2PF imaging results. (**a**), Volumes of molecules. (**b**), Sizes of molecules (hydrodynamic diameters). (**a**) and (**b**), From left to right: fluorescein, FITC-dextran 4K molecule, FITC-dextran 20K molecule, combination of 20K and fluorescein molecules. Scale bars in (**a**) and (**b**): 2 mm. (**c**), Spatially averaged volumes of three types of molecules. (**d**), Spatially averaged sizes of three types of molecules. Error bars in (**c**) and (**d**) represent their standard deviations over space.

Captions of Supplementary Movies

Movie S1. Real-time intensity and polarization anisotropy dynamics of one-photon fluorescence (1PF) from fluorescein molecule in liquid environment, captured by CUP2AI at 125 Gfps. The interval between neighboring frames is 8 ps and there are 316 frames in total. 1PF is excited by a single 400-nm femtosecond pulse with a fluence of 3.1 mJ cm^{-2}. A printed pattern is applied on the cuvette's front face. There are three separate acquisitions, and each row represents the results of one CUP2AI image acquisition. The left two columns are 2D intensity evolutions at both y-polarization and x-polarization. The third column contains 2D anisotropy evolutions and the fourth column plots spatially averaged anisotropy evolving over time.

Movie S2. Real-time intensity and polarization anisotropy dynamics of one-photon fluorescence (1PF) from FITC-dextran 4K molecule in liquid environment, captured by CUP2AI at 125 Gfps. The interval between neighboring frames is 8 ps and there are 314 frames in total. 1PF is excited by a single 400-nm femtosecond pulse with a fluence of 3.1 mJ cm^{-2}. A printed pattern is applied on the cuvette's front face. There are three separate acquisitions, and each row represents the results of one CUP2AI image acquisition. The left two columns are 2D intensity evolutions at both y-polarization and x-polarization. The third column contains 2D anisotropy evolutions and the fourth column plots spatially averaged anisotropy evolving over time.

Movie S3. Real-time intensity and polarization anisotropy dynamics of one-photon fluorescence (1PF) from FITC-dextran 20K molecule in liquid environment, captured by CUP2AI at 125 Gfps. The interval between neighboring frames is 8 ps and there are 304 frames in total. 1PF is excited by a single 400-nm femtosecond pulse with a fluence of 3.1 mJ cm^{-2}. A printed pattern is applied on the cuvette's front face. There are three separate acquisitions, and each row represents the results of one CUP2AI image acquisition. The left two columns are 2D intensity evolutions at both y-polarization and x-polarization. The third column contains 2D anisotropy evolutions and the fourth column plots spatially averaged anisotropy evolving over time.

Movie S4. Real-time intensity and polarization anisotropy dynamics of one-photon fluorescence (1PF) from both fluorescein and FITC-dextran 20K molecules in liquid environment, captured by CUP2AI at 125 Gfps. The interval between neighboring frames is 8 ps and there are 295 frames in total. 1PF is excited by a single 400-nm femtosecond pulse with a fluence of 3.1 mJ cm^{-2}. The cuvettes containing fluorescein and 20K molecules are placed side by side with printed patterns applied on the cuvettes' front faces. The fluorescein and the 20K molecules are on the left and right side, respectively. There are three separate acquisitions, and each row represents the results of one CUP2AI image acquisition. The left two columns are 2D intensity

evolutions at both y-polarization and x-polarization. The third column contains 2D anisotropy evolutions and the fourth column plots spatially averaged anisotropy evolving over time.

Movie S5. Real-time intensity and polarization anisotropy dynamics of one-photon fluorescence (1PF) from both FITC-dextran 4K and FITC-dextran 20K molecules in liquid environment, captured by CUP2AI at 125 Gfps. The interval between neighboring frames is 8 ps and there are 297 frames in total. 1PF is excited by a single 400-nm femtosecond pulse with a fluence of 3.1 mJ cm^{-2}. The cuvettes containing 4K and 20K molecules are placed side by side with printed patterns applied on the cuvettes' front faces. The 4K and the 20K molecules are on the left and right side, respectively. There are three separate acquisitions, and each row represents the results of one CUP2AI image acquisition. The left two columns are 2D intensity evolutions at both y-polarization and x-polarization. The third column contains 2D anisotropy evolutions and the fourth column plots spatially averaged anisotropy evolving over time.

Movie S6. Real-time intensity and polarization anisotropy dynamics of one-photon fluorescence (1PF) from PAH molecule in gaseous environment (kerosene flame), captured by CUP2AI at 50 Gfps. The interval between neighboring frames is 20 ps and there are 128 frames in total. 1PF is excited by a single 400-nm femtosecond pulse with a fluence of 25 mJ cm^{-2}. The height of the imaged region is about 5 mm above the burner. There are two separate acquisitions, and each row represents the results of one CUP2AI image acquisition. The left two columns are 2D intensity evolutions at both y-polarization and x-polarization. The third column contains 2D anisotropy evolutions and the fourth column plots spatially averaged anisotropy evolving over time.

Movie S7. Real-time intensity and polarization anisotropy dynamics of one-photon fluorescence (1PF) from PAH molecule in gaseous environment (kerosene flame), captured by CUP2AI at 25 Gfps. The interval between neighboring frames is 40 ps and there are 103 frames in total. 1PF is excited by a single 400-nm femtosecond pulse with a fluence of 25 mJ cm^{-2}. The height of the imaged region is about 5 mm above the burner. There are two separate acquisitions, and each row represents the results of one CUP2AI image acquisition. The left two columns are 2D intensity evolutions at both y-polarization and x-polarization. The third column contains 2D anisotropy evolutions and the fourth column plots spatially averaged anisotropy evolving over time.

Movie S8. Real-time intensity and polarization anisotropy dynamics of two-photon fluorescence (2PF) from fluorescein, FITC-dextran 4K, and FITC-dextran 20K molecule in liquid environment, captured by CUP2AI at 12.5 Gfps. The interval between neighboring frames is 80 ps and there are 116 frames in total.

2PF is excited by a single 800-nm femtosecond pulse with a fluence of 136 mJ cm^{-2}. Printed patterns are applied on the cuvettes' front faces. There are four samples imaged: fluorescein only, 4K molecule only, 20K molecule only, and the combination of fluorescein and 4K molecules. There is one acquisition for each sample. Each row represents the results of one CUP2AI image acquisition of one sample. In the last sample, the 20K and the fluorescein molecules are on the left and right side, respectively. The left two columns are 2D intensity evolutions at both y-polarization and x-polarization. The third column contains 2D anisotropy evolutions and the fourth column plots spatially averaged anisotropy evolving over time.

References

(1) Wang, P.; Wang, L. V. Single-Shot Reconfigurable Femtosecond Imaging of Ultrafast Optical Dynamics. *Advanced Science* **2023**, *n/a* (n/a), 2207222. DOI: https://doi.org/10.1002/advs.202207222.

(2) Liang, J.; Wang, P.; Zhu, L.; Wang, L. V. Single-shot stereo-polarimetric compressed ultrafast photography for light-speed observation of high-dimensional optical transients with picosecond resolution. *Nature Communications* **2020**, *11* (1), 5252. DOI: 10.1038/s41467-020-19065-5.
