# Supplementary material for: Single-shot two-dimensional nano-size mapping of fluorescent molecules by ultrafast polarization anisotropy imaging
Source: Nat Commun. 2025 May 30;16:5019. doi: 10.1038/s41467-025-60072-1 (PMC12125188; doi:10.1038/s41467-025-60072-1)
Supplement: Supplementary file 2 — Description of Additional Supplementary Files [file 41467_2025_60072_MOESM2_ESM.pdf]

Description of Additional Supplementary Files

Supplementary Movie 1. Real-time intensity and polarization anisotropy dynamics of one-photon fluorescence (1PF) from fluorescein molecule in liquid environment, captured by CUP2AI at 125 Gfps. The interval between neighboring frames is 8 ps and there are 316 frames in total. 1PF is excited by a single 400-nm femtosecond pulse with a fluence of 3.1 mJ cm^{-2}. A printed pattern is applied on the cuvette's front face. There are three separate acquisitions, and each row represents the results of one CUP2AI image acquisition. The left two columns are 2D intensity evolutions at both y-polarization and x-polarization. The third column contains 2D anisotropy evolutions and the fourth column plots spatially averaged anisotropy evolving over time.

Supplementary Movie 2. Real-time intensity and polarization anisotropy dynamics of one-photon fluorescence (1PF) from FITC-dextran 4K molecule in liquid environment, captured by CUP2AI at 125 Gfps. The interval between neighboring frames is 8 ps and there are 314 frames in total. 1PF is excited by a single 400-nm femtosecond pulse with a fluence of 3.1 mJ cm^{-2}. A printed pattern is applied on the cuvette's front face. There are three separate acquisitions, and each row represents the results of one CUP2AI image acquisition. The left two columns are 2D intensity evolutions at both y-polarization and x-polarization. The third column contains 2D anisotropy evolutions and the fourth column plots spatially averaged anisotropy evolving over time.

Supplementary Movie 3. Real-time intensity and polarization anisotropy dynamics of one-photon fluorescence (1PF) from FITC-dextran 20K molecule in liquid environment, captured by CUP2AI at 125 Gfps. The interval between neighboring frames is 8 ps and there are 304 frames in total. 1PF is excited by a single 400-nm femtosecond pulse with a fluence of 3.1 mJ cm^{-2}. A printed pattern is applied on the cuvette's front face. There are three separate acquisitions, and each row represents the results of one CUP2AI image acquisition. The left two columns are 2D intensity evolutions at both y-polarization and x-polarization. The third column contains 2D

anisotropy evolutions and the fourth column plots spatially averaged anisotropy evolving over time.

Supplementary Movie 4. Real-time intensity and polarization anisotropy dynamics of one-photon fluorescence (1PF) from both fluorescein and FITC-dextran 20K molecules in liquid environment, captured by CUP2AI at 125 Gfps. The interval between neighboring frames is 8 ps and there are 295 frames in total. 1PF is excited by a single 400-nm femtosecond pulse with a fluence of 3.1 mJ cm^{-2}. The cuvettes containing fluorescein and 20K molecules are placed side by side with printed patterns applied on the cuvettes' front faces. The fluorescein and the 20K molecules are on the left and right side, respectively. There are three separate acquisitions, and each row represents the results of one CUP2AI image acquisition. The left two columns are 2D intensity evolutions at both y-polarization and x-polarization. The third column contains 2D anisotropy evolutions and the fourth column plots spatially averaged anisotropy evolving over time.

Supplementary Movie 5. Real-time intensity and polarization anisotropy dynamics of one-photon fluorescence (1PF) from both FITC-dextran 4K and FITC-dextran 20K molecules in liquid environment, captured by CUP2AI at 125 Gfps. The interval between neighboring frames is 8 ps and there are 297 frames in total. 1PF is excited by a single 400-nm femtosecond pulse with a fluence of 3.1 mJ cm^{-2}. The cuvettes containing 4K and 20K molecules are placed side by side with printed patterns applied on the cuvettes' front faces. The 4K and the 20K molecules are on the left and right side, respectively. There are three separate acquisitions, and each row represents the results of one CUP2AI image acquisition. The left two columns are 2D intensity evolutions at both y-polarization and x-polarization. The third column contains 2D anisotropy evolutions and the fourth column plots spatially averaged anisotropy evolving over time.

Supplementary Movie 6. Real-time intensity and polarization anisotropy dynamics of one-photon fluorescence (1PF) from PAH molecule in gaseous environment (kerosene flame), captured by CUP2AI at 50 Gfps. The interval between neighboring frames is 20 ps and there are

128 frames in total. 1PF is excited by a single 400-nm femtosecond pulse with a fluence of 25 mJ cm^{-2}. The height of the imaged region is about 5 mm above the burner. There are two separate acquisitions, and each row represents the results of one CUP2AI image acquisition. The left two columns are 2D intensity evolutions at both y-polarization and x-polarization. The third column contains 2D anisotropy evolutions and the fourth column plots spatially averaged anisotropy evolving over time.

Supplementary Movie 7. Real-time intensity and polarization anisotropy dynamics of one-photon fluorescence (1PF) from PAH molecule in gaseous environment (kerosene flame), captured by CUP2AI at 25 Gfps. The interval between neighboring frames is 40 ps and there are 103 frames in total. 1PF is excited by a single 400-nm femtosecond pulse with a fluence of 25 mJ cm^{-2}. The height of the imaged region is about 5 mm above the burner. There are two separate acquisitions, and each row represents the results of one CUP2AI image acquisition. The left two columns are 2D intensity evolutions at both y-polarization and x-polarization. The third column contains 2D anisotropy evolutions and the fourth column plots spatially averaged anisotropy evolving over time.

Supplementary Movie 8. Real-time intensity and polarization anisotropy dynamics of two-photon fluorescence (2PF) from fluorescein, FITC-dextran 4K, and FITC-dextran 20K molecule in liquid environment, captured by CUP2AI at 12.5 Gfps. The interval between neighboring frames is 80 ps and there are 116 frames in total. 2PF is excited by a single 800-nm femtosecond pulse with a fluence of 136 mJ cm^{-2}. Printed patterns are applied on the cuvettes' front faces. There are four samples imaged: fluorescein only, 4K molecule only, 20K molecule only, and the combination of fluorescein and 4K molecules. There is one acquisition for each sample. Each row represents the results of one CUP2AI image acquisition of one sample. In the last sample, the 20K and the fluorescein molecules are on the left and right side, respectively. The left two columns are 2D intensity evolutions at both y-polarization and x-polarization. The third column contains 2D anisotropy evolutions and the fourth column plots spatially averaged anisotropy evolving over time.
