# Supplementary material for: Single-shot two-dimensional nano-size mapping of fluorescent molecules by ultrafast polarization anisotropy imaging
Source: Nat Commun. 2025 May 30;16:5019. doi: 10.1038/s41467-025-60072-1 (PMC12125188; doi:10.1038/s41467-025-60072-1)
Supplement: Supplementary file 11 — Reporting Summary [file 41467_2025_60072_MOESM11_ESM.pdf]

nature portfolio

Corresponding author(s): Lihong V. Wang

Last updated by author(s): Apr 25, 2025

Reporting Summary

Nature Portfolio wishes to improve the reproducibility of the work that we publish. This form provides structure for consistency and transparency in reporting. For further information on Nature Portfolio policies, see our Editorial Policies and the Editorial Policy Checklist.

Statistics

For all statistical analyses, confirm that the following items are present in the figure legend, table legend, main text, or Methods section.

n/a	Confirmed	
☐	☒	The exact sample size (n) for each experimental group/condition, given as a discrete number and unit of measurement
☐	☒	A statement on whether measurements were taken from distinct samples or whether the same sample was measured repeatedly
☐	☒	The statistical test(s) used AND whether they are one- or two-sided<br>*Only common tests should be described solely by name; describe more complex techniques in the Methods section.*
☒	☐	A description of all covariates tested
☐	☒	A description of any assumptions or corrections, such as tests of normality and adjustment for multiple comparisons
☐	☒	A full description of the statistical parameters including central tendency (e.g. means) or other basic estimates (e.g. regression coefficient) AND variation (e.g. standard deviation) or associated estimates of uncertainty (e.g. confidence intervals)
☒	☐	For null hypothesis testing, the test statistic (e.g. F, t, r) with confidence intervals, effect sizes, degrees of freedom and P value noted<br>*Give P values as exact values whenever suitable.*
☒	☐	For Bayesian analysis, information on the choice of priors and Markov chain Monte Carlo settings
☒	☐	For hierarchical and complex designs, identification of the appropriate level for tests and full reporting of outcomes
☒	☐	Estimates of effect sizes (e.g. Cohen's d, Pearson's r), indicating how they were calculated

Our web collection on statistics for biologists contains articles on many of the points above.

Software and code

Policy information about availability of computer code

Data collection	HPD-TA v10.2 software from Hamamatsu was used to acquire images from the streak camera.
Data analysis	Matlab R2023a was used for data analysis. The data processing code was shared on Code Ocean. Code availability statement was included in the manuscript.

For manuscripts utilizing custom algorithms or software that are central to the research but not yet described in published literature, software must be made available to editors and reviewers. We strongly encourage code deposition in a community repository (e.g. GitHub). See the Nature Portfolio guidelines for submitting code & software for further information.

Data

Policy information about availability of data

All manuscripts must include a data availability statement. This statement should provide the following information, where applicable:

- Accession codes, unique identifiers, or web links for publicly available datasets
- A description of any restrictions on data availability
- For clinical datasets or third party data, please ensure that the statement adheres to our policy

The data that support the findings of this study are available from the corresponding author on request. Example dataset is included with the example code deposited in Code Ocean.

Research involving human participants, their data, or biological material

Policy information about studies with human participants or human data. See also policy information about sex, gender (identity/presentation), and sexual orientation and race, ethnicity and racism.

Reporting on sex and gender	This study does not involve human participants.
Reporting on race, ethnicity, or other socially relevant groupings	This study does not involve human participants.
Population characteristics	This study does not involve human participants.
Recruitment	This study does not involve human participants.
Ethics oversight	This study does not involve human participants.

Note that full information on the approval of the study protocol must also be provided in the manuscript.

Field-specific reporting

Please select the one below that is the best fit for your research. If you are not sure, read the appropriate sections before making your selection.

☐ Life sciences ☐ Behavioural & social sciences ☐ Ecological, evolutionary & environmental sciences

For a reference copy of the document with all sections, see nature.com/documents/nr-reporting-summary-flat.pdf

Life sciences study design

All studies must disclose on these points even when the disclosure is negative.

Sample size	This study does not belong to life sciences
Data exclusions	This study does not belong to life sciences
Replication	This study does not belong to life sciences
Randomization	This study does not belong to life sciences
Blinding	This study does not belong to life sciences

Behavioural & social sciences study design

All studies must disclose on these points even when the disclosure is negative.

Study description	This study does not belong to behavioural & social sciences
Research sample	This study does not belong to behavioural & social sciences
Sampling strategy	This study does not belong to behavioural & social sciences
Data collection	This study does not belong to behavioural & social sciences
Timing	This study does not belong to behavioural & social sciences
Data exclusions	This study does not belong to behavioural & social sciences
Non-participation	This study does not belong to behavioural & social sciences
Randomization	This study does not belong to behavioural & social sciences

Ecological, evolutionary & environmental sciences study design

All studies must disclose on these points even when the disclosure is negative.

Study description	This study does not belong to ecological, evolutionary & environmental sciences

Research sample	This study does not belong to ecological, evolutionary & environmental sciences
Sampling strategy	This study does not belong to ecological, evolutionary & environmental sciences
Data collection	This study does not belong to ecological, evolutionary & environmental sciences
Timing and spatial scale	This study does not belong to ecological, evolutionary & environmental sciences
Data exclusions	This study does not belong to ecological, evolutionary & environmental sciences
Reproducibility	This study does not belong to ecological, evolutionary & environmental sciences
Randomization	This study does not belong to ecological, evolutionary & environmental sciences
Blinding	This study does not belong to ecological, evolutionary & environmental sciences

Did the study involve field work? ☐ Yes ☒ No

Reporting for specific materials, systems and methods

We require information from authors about some types of materials, experimental systems and methods used in many studies. Here, indicate whether each material, system or method listed is relevant to your study. If you are not sure if a list item applies to your research, read the appropriate section before selecting a response.

Materials & experimental systems

n/a	Involved in the study
☒	☐ Antibodies
☒	☐ Eukaryotic cell lines
☒	☐ Palaeontology and archaeology
☒	☐ Animals and other organisms
☒	☐ Clinical data
☒	☐ Dual use research of concern
☒	☐ Plants

Methods

n/a	Involved in the study
☒	☐ ChIP-seq
☒	☐ Flow cytometry
☒	☐ MRI-based neuroimaging

Plants

Seed stocks	This study does not involve plants.
Novel plant genotypes	This study does not involve plants.
Authentication	This study does not involve plants.
